# “*I wouldn't survive it, as simple as that*”: Syndemic vulnerability among people living with chronic non-communicable disease during the COVID-19 pandemic

**DOI:** 10.1016/j.ssmqr.2021.100032

**Published:** 2022-12

**Authors:** Josephine M. Wildman, Stephanie Morris, Tessa Pollard, Kate Gibson, Suzanne Moffatt

**Affiliations:** a Population Health Sciences Institute, Newcastle University, Ridley 1 Building, 5th Floor, Queen Victoria Road, Newcastle Upon Tyne, NE1 4LP, United Kingdom; b Department of Anthropology, Durham University, Dawson Building, South Road, Durham, DH1 3LE, United Kingdom

**Keywords:** Syndemic, COVID-19, Non-communicable diseases, Illness work, Social determinants of health, Multimorbidity

## Abstract

The co-occurrence of COVID-19, non-communicable diseases and socioeconomic disadvantage has been identified as creating a syndemic: a state of synergistic epidemics, occurring when co-occurring health conditions interact with social conditions to amplify the burden of disease. In this study, we use the concept of illness management work to explore the impact of the COVID-19 pandemic on the lives of people living with, often multiple, chronic health conditions in a range of social circumstances. In-depth interviews were conducted between May and July 2020 with 29 participants living in a city in North East England. Qualitative data provide unique insights for those seeking to better understand the consequences for human life and wellbeing of the interacting social, physical and psychological factors that create syndemic risks in people's lives. Among this group of people at increased vulnerability to harm from COVID-19, we find that the pandemic public health response increased the work required for condition management. Mental distress was amplified by fear of infection and by the requirements of social isolation and distancing that removed participants' usual sources of support. Social conditions, such as poor housing, low incomes and the requirement to earn a living, further amplified the work of managing everyday life and risked worsening existing mental ill health. As evidenced by the experiences reported here, the era of pandemics will require a renewed focus on the connection between health and social justice if stubborn, and worsening health and social inequalities are to be addressed or, at the very least, not increased.

## Highlights

• Explores syndemic impact of epidemics of non-communicable diseases and COVID-19.
• Uses the concept of illness management work to explore syndemic vulnerability.
• Reveals increased burden of fear of infection and social isolation.
• Reveals amplifying effects of social circumstances on syndemic vulnerability.

## 1 Introduction

The COVID-19 pandemic is co-occurring with epidemics of chronic physical and mental non-communicable diseases (NCDs) such as cardiovascular disease, type 2 diabetes, obesity, and anxiety and depression (Sheldon & Wright, 2020). There is evidence that people with existing physical NCDs are at increased risk of suffering serious harm from COVID-19, with the majority of hospital deaths from COVID-19 occurring in patients with NCDs such as type 2 diabetes, hypertension and ischaemic heart disease (Kluge et al., 2020). There is evidence too of an increase in mental health problems, such as depression and anxiety, during the pandemic due to loneliness, social isolation, and fear of contracting the virus (Khan et al., 2020; Krendl & Perry, 2020). The co-occurrence of COVID-19 and chronic non-communicable diseases has been identified as creating a ‘syndemic’ (Bambra et al., 2020; Mendenhall, 2020): a state of synergistic epidemics, occurring when disease-disease interactions amplify the burden of ill health (Mendenhall, 2017; Singer & Clair, 2003; Singer et al., 2017); for example, type 2 diabetes is identified as one of the most important COVID-19 co-morbidities, operating through a variety of physiological mechanisms to vastly increase the risk of hospitalisation and death from COVID-19 complications (Corrao et al., 2021).

In addition to disease-disease interactions, a vital ingredient of a syndemic is the presence of social factors that enhance vulnerability to disease, further amplify the burden of disease, and complicate the avoidance of disease (Singer et al., 2017). Among the best characterised syndemics are the co-occurrence of HIV/AIDS, substance abuse and violence (Singer & Clair, 2003) and the co-occurrence of type 2 diabetes, poverty, and depression in a number of urban contexts, and among some communities, immigration, violence and abuse (Mendenhall, 2019; Singer & Clair, 2003). In the current pandemic, there are important interactions between COVID-19 and social factors such as socioeconomic disadvantage and systemic racism (Mendenhall, 2020; Sheldon & Wright, 2020).

The threat of COVID-19 creates burdens even in the absence of the disease; that is, suffering is not confined to the infected (indeed, a large proportion of the infected appear to remain asymptomatic (Nogrady, 2020)). There are increasing concerns that the widely adopted public health responses of lockdown, social distancing and self-isolation are themselves impacting on health and wellbeing (Krendl & Perry, 2020; Marroquín et al., 2020). While older people are most vulnerable to the health effects of COVID-19, many younger people are suffering greater psychological and economic impacts from attempts to halt the virus' spread by shutting down large sections of the economy (Belot et al., 2021). Younger people are more likely to be employed in sectors shut down by pandemic restrictions, more likely to have been made unemployed over the course of the pandemic, and are more likely to experience social distancing measures as disruptive (Belot et al., 2021; Costa Dias et al., 2020). Compounding the unequal health impacts of the pandemic, the health-damaging effects of the public health measures are creating a novel form of iatrogenic syndemic (Singer et al., 2017), experienced most severely within disadvantaged communities, where people are more likely to be struggling financially and to access basic resources in ‘lockdown’ (Wright et al., 2020), and are less able to mitigate risks of virus exposure by working from home (Martin, 2021).

To understand how health conditions interact with each other and with social factors to create a syndemic, we need a way of explicating the burdens of disease. The notion that managing the burden of chronic disease often involves “hard and heavy” work has been widely explored (Corbin and Strauss, 1985; May et al., 2014). Illness management requires various types of work: illness-related work, comprising managing both symptom and treatment burdens (e.g., taking medications, following health advice) and the everyday life work (e.g., paid work, looking after home and family) that occurs alongside illness work (Corbin & Strauss, 1985). May et al. (2014) observe that the burden of treatment work takes place within a relational network of support that includes family, friends, and healthcare professionals.

The work of managing multi-morbidity is particularly challenging due to the requirement to cope with a range of physical, emotional and social experiences (Coventry et al., 2015). Illness trajectories are constantly shifting, to a greater or lesser degree, and each trajectory change requires changes in the type and nature of work and the resources required to perform it. Uncertainty and flux are features of life with co-morbidity (Coventry et al., 2015). The concept of the work required for chronic illness management has parallels with the concept of a syndemic in that the work of managing physical ill-health is amplified by the presence of co-morbidity, depressive illness, and socioeconomic disadvantage (O'Brien et al., 2014). People living with disease “exist at the intersection of social, personal, and clinical circumstances” (Shippee et al., 2012, p. 1042). Disruptions to an illness trajectory, such as those caused by worsening health, the onset of a new morbidity, a deterioration in social circumstances, a disruption to a relational network – or a global pandemic – can make management more difficult by throwing routines into disarray, unbalancing workloads and creating a “domino effect”; a downward spiral of overwork, fatigue, de-motivation, depression and loss of control (Corbin & Strauss, 1985, p. 239). In this study, we aim to use a syndemics framework to explore the impacts of the COVID-19 pandemic on the lives of people living with, often multiple, chronic health conditions in a range of social circumstances. Qualitative data provide unique insights for those seeking to better understand the “consequences for human life and wellbeing” (Singer et al., 2017, p. 942) of the interacting social, physical and psychological factors that create syndemic risks in people's lives.

## 2 Methods

### 2.1 Study context

This study was set in a city in North East England. Participants were part in of an ongoing study (commenced in 2018) investigating the impact on health-related quality of life of a community-based social prescribing intervention, targeting patients aged between 40 and 74 years with a diagnosis of at least one of a range chronic health conditions including cardiovascular disease, type 2 diabetes, and depression or anxiety (Moffatt et al., 2019). Patients are referred from primary care to a link worker who helps them to access sources of community support to address health behaviours and the wider social determinants of health.

During the current study, England's population was living under changing government COVID-19 lockdown restrictions, including a national lockdown between March 22 and June 1, 2020. One million clinically extremely vulnerable people were advised to ‘shield’ in their homes for 12 weeks from March 22, 2020, avoiding all in-person contact with others (Institute for Government, 2021). The ‘stay at home’ restrictions (March 23, 2020 to May 12, 2020) directed everyone to remain at home, including, where possible, to work from home, throughout this period except for a limited number of ‘essential’ reasons (Institute for Government, 2021). To help limit job losses, the UK government introduce a Coronavirus Job Retention Scheme on March 20, 2020, enabling employers to furlough staff and for a government grant to cover 80% of staff wages (up to £2500 per month) (Department for Work and Pensions, 2020). To support those made unemployed due to the pandemic, the UK government also announced a temporary 12 month £20 per week increase in the rate of Universal Credit, the UK's main working-age welfare benefit (Mackley et al., 2021). The increase was subsequently extended by five months, ceasing in October 2021.

From May 13, 2020, restrictions were gradually relaxed to allow more outdoor mixing and the ‘stay at home’ message was replaced with the, more ambiguous, ‘stay alert’ message (Institute for Government, 2021). From June 13, 2020, support bubbles allowed single adult households to meet with members of one other household. Restrictions were further eased through June 2020, with a phased schools reopening, meeting six people outside permitted and restrictions on leaving home replaced with a requirement to be home overnight (from June 1, 2020), the re-opening of non-essential shops (June 15, 2020) and a relaxing of social distancing rules from 2 to 1 ​m (June 23, 2020) (Institute for Government, 2021).

### 2.2 Data collection

Participants in the on-going social prescribing study had completed a baseline health-related quality of life questionnaire in the months July 2018 to June 2019. At the time of this present study (May to July 2020), participants were being re-contacted to collect 12-month follow-up data. From May 2020, after they had completed the survey, participants were invited to take part in a telephone interview about their experiences of the pandemic. Sixty participants were contacted with a request to complete a follow-up questionnaire between May and June 2020 and 29 agreed to take part in an interview.

Prior to interview, participants were posted an information sheet and consent form. Interviews were conducted between May 11 and July 13, 2020 by SLM, TP, KG and SM. Consent was verbally audio recorded prior to the interview commencing. Interviews were framed around a topic guide, which covered participants and their household members' health, shielding status and direct experiences of COVID-19; and the impact of the pandemic on everyday life, employment, health and relationships. Demographic data were collected on age, gender, ethnicity, employment, education, household income, and housing tenure/type/composition. At the start of the interview, participants were asked to complete a further questionnaire to collect data on their current health-related quality of life. Health-related quality of life was measured using the EQ-5D-5L, which captures quality of life across five domains of mobility, self-care, usual activities, pain/discomfort and anxiety/depression (EUROQOL Instruments, 2017). Respondents rank their current state in each domain on a scale of 1 (no problems) to 5 (unable to perform or extreme problems). A person reporting no problems in any of the five domains would have a health state of 11111, while someone reporting extreme problems in all domains would have a health state of 55555. One participant declined to complete an EQ-5D-5L questionnaire.

Interviews were audio recorded and lasted between 20 and 90 (average 44) minutes.

### 2.3 Data management and analysis

Interviews were professionally transcribed, checked by the research team for inaccuracies and anonymised. NVivo 12 supported data management and coding. A thematic approach to data analysis was taken (Braun & Clarke, 2006). Transcripts were read by all authors allowing for immersion and familiarity. Data analysis was led by JMW and SLM who conducted close reading and re-reading of the transcripts. Initially a priori and inductive coding frameworks were developed using line-by-line coding by JMW and SLM and discussed with the whole research team before being applied to the transcripts. The coding framework was refined using constant comparison to develop conceptual themes. To ensure rigor, all transcripts were independently coded by both JMW and SLM. Discrepancies in coding were discussed and resolved. All authors met regularly to discuss emerging themes and develop the final analysis. Names used in this paper are pseudonyms and identifiable personal details have been omitted. We present interview participants' EQ-5D data collected at interview to characterise their health-related quality of life.

## 3 Results

**Fig. 1:**
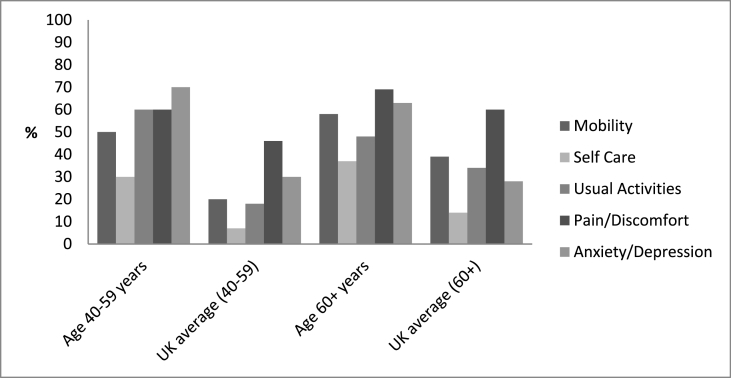
Proportions of participants reporting a problem (score of 2 or more) in each of the EQ-5D-5L domains.

Participant characteristics are presented in Table 1. Median age was 64 (range 42–75 years). The mean number of chronic conditions was 2.76 (ranging between one and nine). The majority of participants were White British; three were British Bangladeshi/Pakistani/Indian. Seven participants lived alone, 17 lived with a spouse or partner, three lived with a partner and school-age children, and two were single parents living with school-age children. Twenty participants were living in households with lower-than-median incomes (median household income in 2020 was around £30,000) (Office for National Statistics, 2020). Seven participants were in employment, 15 were receiving welfare benefits (e.g., Universal Credit), and 16 were retired from paid work (some had taken early retirement due to their health). Four participants reported receiving official advice to shield, while two were living with family members advised to shield. Fig. 1 illustrates the state of participants' health-related quality of life, with reference to the UK average (Janssen & Szende, 2014). Participant's EQ-5D-5L data demonstrate that, compared to the English average values for each domain, health related quality of life was poor for both younger (aged 40–59 years) and older (aged 60 and over) participants. Health-related quality of life scores varied widely between participants (Table 1) with some reporting no problems in any domain, while others reported problems, some severe or extreme, in most or all domains.

**Table 1:** Participant characteristics.

Gender		N ​= ​29 (%)
	Male	13 (44)
	Female	16 (56)
Age (years)		
	40–49	4 (14)
	50–59	6 (21)
	60–69	13 (44)
	70+	6 (21)
Ethnicity		
	White British	26 (90)
	Bangladeshi/Pakistani/Asian Punjabi	3 (10)
Occupational status		
	Employed	4 (14)
	Employed - furloughed	1 (3)
	Employed - shielding	3 (10)
	Unemployed	5 (17)
	Retired	16 (56)
Household income (£)		
	<10K	8 (28)
	10–20K	7 (24)
	21–30K	5 (17)
	31–40K	3 (10)
	>40K	2 (7)
	Prefer not to say	4 (14)
Benefits claimed^a^		
	None	14 (49)
	Attendance or carers allowance	5 (17)
	DLA/PIP, ESA, LCW	5 (17)
	Universal Credit, child tax credits	5 (17)
Household structure		
	Lives with partner/family	22 (76)
	Lives alone	7 (24)
Number of non-communicable conditions		
	1	5 (17)
	2	10 (34)
	3	6 (21)
	4 or more	8 (28)

a Attendance allowance is available for people of pension age or older with a physical/mental disability severe enough to require care; carers allowance is available for people who provide care for 35+ hours a week; child tax credit is available for people responsible for raising a child (up to age 16, or age 20 if that child is in full time education or training); PIP (Personal Independence Payment) is replacing DLA (Disability living allowance) for working-age people with a disability; ESA (Employment Support Allowance) is available for people with a disability or health condition that affects their work capacity; Universal credit (UC) is available for people on a low income, out of work or unable to work.

Findings are grouped into three themes: firstly, the mechanisms through which COVID-19 interacted with chronic health conditions to increase the work required for illness management; secondly, the amplifying effects on existing mental distress of fear of infection and the requirements of social distancing; and thirdly, the further amplifying effects of social conditions, such as poor housing, low incomes and the requirements to earn a living, on the work of managing everyday life during the pandemic. Key participant characteristics are provided in the form: [age group_employment status_family circumstances_EQ-5D domain scores: mobility, self-care, usual activities, pain/discomfort, anxiety/depression (1 ​= ​no problems, 2 ​= ​slight, 3 ​= ​moderate, 4 ​= ​severe, 5 ​= ​extreme problems)].

### 3.1 Synergistic impacts on illness management work

A syndemic framework requires an exploration of how health conditions are experienced by people, including in terms of somatic experiences and daily activities (Mendenhall, 2017). Interview data allow us to explore the ways in which the co-occurrence of non-communicable disease and the risk of infectious disease amplified participants' existing symptom and treatment burdens. Participants’ accounts revealed the work involved in living with multiple chronic conditions. Illness management work for many involved managing complex medication regimens and maintaining mobility, undertaking usual activities and performing self-care tasks - the functional domains of health-related quality of life - required planning and adaptation.

An individual's ability to manage their health requires the capacity, or agency, to engage with healthcare services, with engagement determined by the availability of services (May et al., 2014). Among many participants, COVID-19 restrictions were creating practical challenges for performing illness work, with the pandemic response reducing or removing aspects of control over illness management. In an epidemic, in the face of limited resources, decision-makers respond by prioritising one disease over others, usually the disease that poses an immediate threat to population health. The healthcare system's prioritisation of COVID-19 created difficulties for accessing healthcare for existing NCDs. Rafi [40–49_furloughed_living with wife and young children_21222] explained:

*It's so difficult to make an appointment. There are times when I would like to go to the doctor. It's not an emergency but I would like to go there for reassurance, but honestly, the conditions and criteria, it's absolutely horrendous. First, you need to get through to the phone. And when you manage to get through to the phone, someone picks the phone up, they ask you hundreds of questions for whatever reason, then, “Sorry, there is no appointment today, can you ring back tomorrow morning at 8:00am?” And then when you ring back, you're in a queue. It is endless.*

The prioritisation of COVID-related healthcare was removing opportunities for lessening the burden of illness work through the collaborative self-care (Yin et al., 2020) usually performed alongside the healthcare providers within the relational networks that support self-care (May et al., 2014). Losing opportunities for routine monitoring and seeking reassurance made “*knowing where you were*” with condition management more difficult, even for those reporting better health-related quality of life. Heather [60–69_retired_living with partner_11121] described her frustration at being left to monitor her condition alone:

*I haven't had anything. Fair enough, I know there's been a problem, but when you read up about [my condition], it is saying how you should have a six-monthly review and they should be checking this and checking that. Well, they haven't and I'm three months overdue … I'm thinking, ‘How do they know how my [condition] is getting on and how I am feeling?’ They don't know.*

May et al. (2014) identify ‘reflexive monitoring’ – self-surveillance - as a form of patient work. Participants' accounts made visible the work involved in the invisible tasks of condition management, such as exercising willpower and resisting temptation (Yin et al., 2020) within disrupted healthcare support networks. Conditions responsive to self-care in the form of lifestyle behaviours, such as diet and exercise, were understood to require illness-related work in the form of shouldering responsibility for self-surveillance and “*being very strict with myself*” [Jessica: 40–49_employed_living with partner and children_11112]. However, exercise, which participants recognised as important for keeping physically and mentally well, was made more difficult or impossible by lockdown restrictions that had resulted in the closure of gyms and leisure centres and restrictions on going outdoors. Maintaining the motivation for self-care while struggling with boredom and low mood was creating additional illness work, leading to ‘failure’ for some participants. Poor diet plays a role in the aetiology of many NCDs and is itself closely associated with socioeconomic disadvantage (Darmon & Drewnowski, 2015). Many participants were struggling with their weight and were very aware that their chronic conditions were, in part, both caused and exacerbated by poor diet. However, pandemic conditions made efforts to eat well that much harder. Derek [50–59_employed_living alone_22333] was struggling to manage one of his chronic conditions. He explained:

*It will be because I'm eating chocolate and it's all sugar-based … it's boredom eating, when you think about it, because there's nothing else you can do. You can only watch so much telly, watch so many DVDs, read so many newspapers. What else can you do? I just comfort eat. That's all got to stop. It's going to have to stop, because I'll just make myself worse.*

Like Derek, Reena's [50–59_employed_shielding_living alone_11113] “*comfort eating*” was also affecting her weight. She identified the invisible work of maintaining self-control and motivation as becoming “*harder and harder*” as lockdown continued.

Co-occurring diseases are necessary but insufficient conditions for a syndemic: also required are amplified health consequences of synergistic disease interactions (Singer & Clair, 2003). For some participants, the inability to perform their usual illness work was exacerbating the symptoms of their chronic conditions. Martin [70–79_retired_living with wife_44443] reported very poor health-related quality of life. COVID-19 restrictions had closed his local gym and his inability to attend his regular session were a “*big, big miss … [I] can't wait to get back*. *I can feel the difference in me. I'm not so fit. My chest is tighter. I am on the inhalers more and things like that*”. For some, functional aspects of health-related quality of life were complicated by pain that was a constant, debilitating presence in many participants' lives. Pain complicated everyday life work and the effort required to keep it at manageable levels was increased under pandemic restrictions. For participants, like Derek, who were living with pain or discomfort, enforced inactivity was worsening suffering and heightening the need for reflexive monitoring:

*It [pain] has been getting worse because, with me not walking about and getting the circulation going. As you know, if you sit down long, your legs get dead stiff. If I go on an aeroplane and you've been on a plane for hours and you get off, and you've got people dying through blood clots in their veins and stuff. So, I'm just wary with that, you know?*

Within their relational networks, some participants held the roles of both patient and carer for a family member with chronic ill health. While Martha [60–69_retired_living with husband_11112] felt the impact of the pandemic on her own health and wellbeing was minimal, her struggles stemmed from the impact on her vulnerable husband. Enforced inactivity from the ‘stay at home’ directive was worsening his condition and impacting on Martha's own wellbeing and her efforts to remain cheerful. She explained that, *“before lockdown, we used to go out quite a bit … [his symptoms worsened] when he wasn't being occupied, so it has been worse during lockdown. I am not sleeping and I am irritable. I try not to, but … ".*

The additional efforts required for caring increased the syndemic burden of people living with their own chronic ill health.

### 3.2 The amplification of mental distress

#### 3.2.1 Fear of infection

A particularly burdensome aspect of an infectious disease pandemic is the fear it provokes (Brown and Marí Sáez, 2020). Participants had become, to various degrees, accustomed to living with non-communicable diseases. An infectious and potentially lethal disease presented a new threat to life. A further necessary condition of a syndemic is the risk of enhanced vulnerability due to disease interactions (Singer & Clair, 2003). An acute awareness that their NCDs placed them at increased vulnerability to serious harm from COVID-19 was causing deep anxiety for many participants - as Martin bluntly stated: *“I'm one of the dangerous ones … I wouldn't survive it. As simple as that”*. Fear creates additional cognitive burden, with anxiety not confined to personal risk; many were also anxious for their loved ones. Rosalind [70–79_retired_living with husband_21122], like Martha, was caring for her husband and her fears centred on his vulnerability: *“because my worry is if I go out, I'm not saying that I will get it, but if I bring it back in, my husband would never survive. I could never forgive myself for that"*.

The burden of illness is amplified when illness work and everyday life work are in tension with each other (Corbin & Strauss, 1985). Some participants were too frightened to go about their daily lives while, *“there is something terrible out there”* [Reena]. In their study of the Ebola virus epidemic in West Africa, Brown and Marí Sáez (2020), describe the sense of threat from “a mobile, invisible enemy that could be anywhere”. Our participants, too, experienced COVID-19 as *“an invisible killer”* [Derek]; it was this invisible threat that kept Reena scared to leave her home:

*I'm frightened of catching it, because I know that if I get this virus it will kill me, because my heart is not strong. My chest is bad, and I know it will kill me. I'm really scared to go out … If somebody said to me today, “Today you can go out.” I don't think I could. I'm scared. You can't see it, can you? You don't know where it is, do you, this virus? It could be anywhere …*

Psychological distress can reduce an individual's capacity to access care (Shippee et al., 2012). Fear of the virus was complicating illness work for William [60–69_retired_living with wife_44443] who was too frightened to access the healthcare he needed to cope with his multiple chronic conditions. William was adamant that:

*I won't go to the doctors. I should really, but I won't go. I just don't think it's safe for now. I worry if I go to the doctors … I should really go to the doctors, but I won't go because I wouldn't feel safe going to the hospital. I mean, I've worked there, I know what it’s like there.*

Again, there are parallels with Brown and Marí Sáez' (2020) descriptions of the Ebola virus epidemic, where medical staff and treatment centres, previously sites of comfort and support, became sites of fear.

A feature of the COVID-19 pandemic is the emotional burden created by the necessity of relying on other people to minimise risk through collective sacrifice. A new dimension to illness work had been created by the need for a collective societal effort – a dramatic expansion of relational networks - to help the vulnerable to stay safe. ‘Rule breaking’ and the failure of others to play their part in this new form of collaborative self-management was eroding the capacity to avoid infection and creating an emotional state that Jessica described as, *“somewhere between angry and extreme anxiety”*. Other people became potential sources of infection and were viewed with a degree of suspicion. Brown and Marí Sáez (2020) draw parallels between the public health response to the West African Ebola virus epidemic and the COVID-19 response, with both resulting in “a deliberate and sudden undoing” of social relations that altered people's ability to trust others. Sarah [60–69_retired_living alone_33332] expressed that illness work would be increased by the breakdown of trust in the presence of an invisible threat:

*Well, I think everybody is going to be not trusting each other and I think we're going to be scared to cough. It's like one of these horror films, they'll be pointing at you. They won't be doing it physically, but mentally you'll be thinking, “God, everybody …“. It makes you feel a little bit strange, I think. You know, you'll not be trusting people. You'll not want nobody to sit beside you on a bus and things like that. It is going to be different. It's not going to be the same like before. People aren't going to trust each other anymore when they go out, I don't think.*

The sense of unreal menace created by the pandemic from Sarah's ‘horror film’ analogy was described in very similar terms by other participants.

#### 3.2.2 Loneliness and social isolation

While for some, the mental impacts of the pandemic were mitigated by emotional support from close family units, for others the relational networks providing vital support with illness work had been severely disrupted. Although acknowledged as necessary, the social distancing required to stay safe from COVID-19 was having by far the greatest impact on mental health. Loss of the sociability of work was causing mental distress for some who were accustomed to going out to work every day. John's [50–59_employed_shielding_living with partner_21331] health condition required he shield, but he was desperate to get back to work. He explained: *“I can't wait. Honestly, it is soul destroying, just sitting here all day. You're used to having to get out and talk to people”*. Similarly, Beverley [50–59_employed_living with husband_43445] identified home-working as impacting on her already poor mental health:

*I think sometimes the depression it affects that because I'm used to being in an office with people, so being at home on your own is quite difficult when it's day after day … I think it's just missing the routine … I'm still trying to get up and do my job early in the morning, so if my husband is on a day off like today and it fairs up we can go and sit in the garden or something. But there's not really much to get up for work early though because the afternoons, it's exactly the same.*

Chronic physical conditions were co-occurring with chronic mental conditions among many participants and the pandemic was worsening existing mental distress. The disruption of relational networks meant that previously shared burdens had to be shouldered alone. Social isolation was leaving some vulnerable to intrusive thoughts. As Derek observed, *“it's horrible being on your own, because your mind wanders off and you think bad things”*. For Martin, keeping busy was a tried-and-tested strategy for keeping distressing thoughts at bay. The constraints of lockdown were making distraction work harder to perform:

*Your mind, it is not active enough. It is drifting back to past things that have haunted you … it is something you can't move. You can't just say, “Forget it.” There are two or three things that happened and, like I say, sets me back. That's when you have got too much time on your hands and you are sitting. If you are active and you're doing things, your mind has not time to go back to things.*

Much-referenced by all participants struggling with social isolation was a strong desire for physical contact. A yearning for human touch was particularly acute for those living alone, like Amanda [50–59_receiving sickness benefits_living alone_33434] who was coping alone with a health crisis made much harder in the absence of her usual sources of comfort:

*It is awful. You get very low at times, to where you're reduced to tears and then I have grandchildren as well and I see them on WhatsApp, but I just want to huggle them, you know? It's ridiculous. I miss face to face contact. I understand why it has to be done, but it's the physical thing.*

As Brown and Marí Sáez (2020) note, the challenge of caring for loved ones while observing the requirements to protect the wider collective is “partly what makes epidemics so traumatic”. The pandemic was increasing the emotional work of caring among participants providing support to loved ones. Remote forms of contact were desperately inadequate in the face of suffering. William described his distress as lockdown prevented in-person contact with his much-loved - and vulnerable - sister:

*I haven't seen my family at all. It really gets us down, depressed. I'm very close to my sister … I just worry about her, but I cannot see her. I've rung her, but I don't like to ring her too much because she starts crying on the phone. It's hard, you know. She's taken it really bad … I'm finding it hard myself, it really gets you down. Sometimes you're sitting here with yourself and you think, “I may as well just go ….” I feel … like I say, if you don't feel right in yourself, you know what I mean, what's the point?*

It is difficult to balance the benefits of reducing viral transmission against the harms of social distancing. Gill [60–69_receiving sickness benefits_living alone_31135] described the deeply distressing experience of attempting remote contact with her husband who was resident in a care home:

*I couldn't go in, at all. He didn't understand. I used to ring him on the telephone and he would say, “Where are you? I am waiting for you to come. What are you not coming for?” That was very distressing for me, and it was also distressing for him because he didn't understand why all of a sudden I had stopped going to see him. But then, as the time went on, and on, him saying things like that just started to stop. I think he had just realised that I wasn't going to go back to see him, in his own mind, and he started to deteriorate from then on. And I think it was all down to me not getting in to see him. I was the only person he really recognised, and that was taken away from him. It was taken away from me, as well, which was really hard.*

While fear of infection, loneliness and the trials of socially distanced relationships affected many participants, Gill had been directly impacted by infection, losing her husband to COVID-19. From the point of his diagnosis, lockdown measures had created a deeply traumatic series of events that culminated in Gill enduring a socially distanced funeral:

*… I got the phone call to say he had passed … And I couldn't be with him. And I did ask if I could go in on that day and they said, “No.” So, it is so hard that I wasn't with him at the end when I should have been. And it has just been absolutely devastating. And then when we had his funeral … because I live on my own and everybody else was his family. They were all in groups, and I was just the only one standing there. So, I couldn't have any comfort from anybody and I found that dreadfully, dreadfully hard.*

The requirement to *“be strong”* while unable to give - or to receive - comfort while she was widowed had devastated Gill's mental health. There is a limit to how long people can carry on in often very difficult circumstances. The cognitive work required to cope with radical upheaval consumes already limited resources and is unlikely to be unsustainable long-term (Yin et al., 2020). Many participants ended their accounts with a deeply expressed desire for life to *“get back to normal”* as quickly as possible. Long-term illness management requires hope and the possibility of a reward for effort expended (Corbin & Strauss, 1985). While Derek was hoping that, *“something good has got to happen”*, he had:

*… just got a funny feeling it's going to all start all over again. I don't want that to happen, because I wouldn't be able to think straight then. If I was locked in again for another three to four months. I think I'd get really depressed then.*

Subsequent to this study's data collection period, England experienced two further periods of nation lockdown (November 5, 2020 and January 6, 2021).

### 3.3 Syndemic effects of social conditions

A syndemic framework captures the ways in which social conditions interact with health. Some participants, including some with poor health-related quality of life, felt they were coping well in the pandemic under the circumstances, with access to the resources needed to adapt and with minimal disruption to their routines or illness trajectories.

Participants who were retired from paid work and living on fixed incomes reported experiencing no financial disruptions from the pandemic; indeed, some were enjoying spending less and saving money. For a few working-age participants, a less hectic life freed from demanding everyday life work was making it easier to manage long-term conditions, creating space for self-care and pursing more enjoyable activities. The requirement to shield had freed Sadiq [50–59_employed_shielding_living with wife and children_11112] from his gruelling work schedule to enjoy time with his children.

*I don't feel any boredom or anything. I feel really good rather than working. Oh my goodness, it's like a long time holiday. It doesn't affect the relationship, that's very good … All are happy. They don't think about anything. We don't worry about it, the situation, because we're staying home all the time. It helps we are all quite well. There are no jobs, no work, just eat and drink and be merry, that's it.*

Sadiq's wife was in full-time employment and the family were managing on her wage.

However, Sadiq's experience was atypical; for many, the pandemic had added to the multiple complicating factors that increased the burdens of illness work (Shippee et al., 2012). Participants struggling most with COVID-19 restrictions tended to be those of working age, without the option of remaining safe at home or already struggling on low incomes or in poor housing. Derek described the restrictions on social contact imposed by his housing conditions:

*I've even got to stay away from my neighbour who's directly opposite. There's a 4-foot to 5-foot passageway. There are six flats on every floor, so even the lad across the landing, I can't even talk to him, because he's within the 6-foot boundary.*

For some participants in paid work, everyday life work required for employment during the pandemic was in tension with the illness work required to maintain health. Concern about bringing the virus home to his vulnerable family had caused Jessica's husband to stop his work as a professional driver. She explained:

*[He] was quite mindful that he didn't want to be putting the family at risk. At the beginning, before lockdown happened, he was working then, and was saying to people, “Can you sit in the back?” And people were just point-blank refusing; they were just like, “Why?” and really questioning it. And he was just saying, “Just for both of our safety … ". People were just not … So yes, he made a decision … that he was not going to work, just to protect us as a household.*

The family were trying to manage on one rather than two wages.

The capacity to undertake illness work is eroded by socioeconomic disadvantage (May et al., 2014). The social conditions that put people at increased risk of NCDs were also those conditions that meant they were less able to avoid infection, or paid a higher price for doing so. The option to work at home was unavailable to some participants. James [60–69_living with wife_employed_21231] was working in retail. Although his work had become harder during the pandemic, he felt his efforts were unvalued by his employer:

*I'm standing in front of customers. I'm chasing shoplifters and everything. Keeping people safe and I get a load of rubbish off them as well. Nobody thinks about safety for us. This has been going on for how long now? 14 weeks now, roughly? Believe it or not, our office has just sent us masks out in the last week.*

James' employer had also reduced his hours, causing him financial stress. The economic impacts of the COVID-19 pandemic have been unequally distributed, with people already on lower incomes suffering more financial effects than the more affluent (Bambra et al., 2020; Belot et al., 2021). Practical measures, such as the furlough scheme, are to be welcomed for allowing vulnerable people to stay safe. However, for those already struggling to get by, furlough was no panacea. Although the scheme replaced 80% of Rafi's income, an already low income made the everyday life work of budget balancing impossible:

*The money has just come through about two weeks ago, but I was in debt, I'm still in debt. When you get a very small wage packet, and you get a reduction of 20% … If I had a big wage packet, 20% would have been okay, but when you're on a very small wage packet to start off with, and you're getting reduced by 20%, you're not left with much … We had to reinvent our everyday life, expenditure and everything. But still it's a struggle. When you've kids in the house, it's difficult for them to understand why we have to change certain things, to do differently … it's been a massive impact on the way we can run our daily lives.*

Rafi spoke little about his health; instead it was financial uncertainty that was *“killing”* him:

*We don't even know if we're going to have a job at the end of the day. So the uncertainty is … That's what killing me at the moment. I think, when I mentioned just before about anxiety, obviously you get a bit depressed, not knowing … a bad situation is not knowing what's going to happen in the future, the uncertainty. So anxiety just creeps in … the biggest worry is the uncertainty. After lockdown, how it's going to be? What if I have the same, in the way we are living? Then what is going to happen? It's the uncertainty, that's what's killing me. Because I've got two kids and a wife to look after. It's still finance; it's really to do with the finances. Even if I wanted extra hours now, I can't get any extra hours. Even when furlough is gone, there are no extra hours. There's even a chance there might not be a job there.*

For those of working age but without work, the conditionality of social security was a source of anxiety. In response to the COVID pandemic, welfare benefit conditionality, including obligations to attend appointments with advisors and provide evidence of actively searching for work, were suspended in March 2020. Conditionality was re-introduced in July 2020. The work required to comply with welfare conditions, for Jude [60–69_unemployed_living alone_43443], was in conflict with avoiding infection, with the threat of sanctions for non-attendance of in-person appointments weighed against her fear of exposure to the virus on public transport:

*It would be about five years [since I worked], I think. But I had real health problems. At the start of it, they found out what was wrong with me, and I wasn't doing very well. But now I'm monitored on tablets, they want me to really try and get a job, when this is- The Job Centre, yes … I've taken them to a tribunal and I lost it … that was a couple of months- Just before it [pandemic] all happened in March … So, they think I should be looking for a job, a part-time job. Well, I've got to go to the Job Centre, to go and see them, and I've got to go on- I haven't been for the last three months, since they've had the lockdown, but my appointment is for June, so I'm going to have to go on the bus. I'll be dreading that … because I'm a bit frightened to go … it's just feeling close, the closeness. I just don't feel safe. [But] if you don't go, they'll take your money off you.*

Syndemic vulnerability was greatly increased for participants whose chronic ill health placed them at increased risk of harm or death from COVID-19, but who lacked the resources to comfortably shield themselves and their loved ones from harm. The long-term effects of debt and depression are likely to outlast the effects of the pandemic.

## 4 Conclusions

Anything that erodes capacity to perform illness work increases the burdens of condition management (May et al., 2014; Shippee et al., 2012). The COVID-19 pandemic has created a far from “ideal context” (Corbin & Strauss, 1985, p. 230) for illness management. This study's qualitative data offers rich insights into the syndemic effects of the COVID-19 pandemic within the lives of people living with chronic health conditions. Beyond the co-occurrence of diseases, a syndemic requires the presence of excess disease burdens, further amplified by social conditions (Singer & Clair, 2003). We identify ways in which illness work and the everyday life work required to manage health are made practically and psychologically much more difficult by the threat of the COVID virus, by the public health responses of self-isolation, social distancing and lockdown, and by social factors such as poor housing, low income and the need to earn a living. Shippee et al. (2012) identify a feedback loop of increasing patient workload that occurs when an increase in the burden of symptoms, treatment and everyday life work reduces a patients' capacity to access healthcare or carry out self-care, and leads, inevitably, to worse outcomes. Our findings provide an illustration of a feedback loop that typifies a syndemic: social conditions predispose people to co-occurring physical and mental NCDs and to increased risk of COVID-19; social conditions interact with co-occurring diseases to increase the effort required to manage health, while also reducing the resources available to do so. Inevitably, all too often, the result is a cycle of worsening health and diminishing capacity to stay well. For some of this study's participants, disruptions to the balance of effort required to fit together illness-related work and everyday life work, and a lack of resources that would help them adapt, resulted in a ‘domino effect’ of worsening physical and mental health (Corbin & Strauss, 1985).

For many years now, there has been awareness and acknowledgement of the links between social factors and health. Efforts to address the social determinants of health, however, have been ineffectual and underwhelming (Allen & Feigl, 2017; Marmot et al., 2020), with the unequal impacts and economic costs of the epidemic of NCDs yet to result in meaningful action by governments in countries like the UK (Sheldon & Wright, 2020). The era of pandemics (United Nations, 2020) will require a “renewed focus on the connection between health and social justice” (Singer & Clair, 2003, p. 431). Mendenhall (2020) has taken issue with the notion that COVID-19 is part of a pandemic syndemic, arguing that such a characterisation overlooks the differing socioeconomic contexts and political responses that serve to amplify the burden of disease. For example, there is emerging evidence that countries that have experienced higher rates of COVID-19 cases and deaths have higher levels of income inequality that “is a proxy for many elements of socioeconomic disadvantage that may contribute to the spread of, and deaths from, COVID-19" (Wildman, 2021, p. 456). The catastrophic economic effects of the pandemic provide a persuasive case for addressing socioeconomic inequalities, but there is as yet scant evidence that UK government policy is addressing these in any meaningful way (Marmot et al., 2020). Indeed, the UK government has removed the £20 per week pandemic-related Universal Credit uplift, which had been found to represent a significant share of welfare entitlements for many claimants, particularly those for whom Universal Credit is their only source of income - a group that has increased in size due to the pandemic (Mackley et al., 2021).

Our study has several limitations. Participants were all living in a city in North East England, which at the time of the study, had relatively low rates of COVID-19 cases. Experiences of the pandemic may have been different in areas with higher case rates. Further, North East England is among the least ethnically diverse regions of England; nearly 94% of people living in North East England are White British (Office for National Statistics, 2018). As people from ethnic minority backgrounds appear to be at increased vulnerability to COVID-19, their accounts of the syndemic impacts of the pandemic may differ. Social distancing regulations meant that interviews were conducted by telephone. Face-to-face interviews may have allowed participants to provide more detailed accounts; however, our experience was that participants were happy to be interviewed remotely. Indeed, a number commented they found it easier to talk about their experiences at a distance. Finally, interviews were conducted in the first few months of the pandemic, during which time little was known about the how long the pandemic would last. It is possible that participants' responses may have changed over time. Longitudinal interviews would have better captured evolving experiences.

## Ethics statement

Ethical approval was gained from Durham University Ethics Committee.

## Declaration of competing interest

The authors declare that they have no known competing financial interests or personal relationships that could have appeared to influence the work reported in this paper.
